# Brain and Cognition for Addiction Medicine: From Prevention to Recovery Neural Substrates for Treatment of Psychostimulant-Induced Cognitive Deficits

**DOI:** 10.3389/fpsyt.2019.00509

**Published:** 2019-07-24

**Authors:** Manoranjan S. D’Souza

**Affiliations:** Department of Pharmaceutical and Biomedical Sciences, The Raabe College of Pharmacy, Ohio Northern University, Ada, OH, United States

**Keywords:** cocaine, nicotine, methamphetamine, memory, extinction, nucleus accumbens, prefrontal cortex

## Abstract

Addiction to psychostimulants like cocaine, methamphetamine, and nicotine poses a continuing medical and social challenge both in the United States and all over the world. Despite a desire to quit drug use, return to drug use after a period of abstinence is a common problem among individuals dependent on psychostimulants. Recovery for psychostimulant drug-dependent individuals is particularly challenging because psychostimulant drugs induce significant changes in brain regions associated with cognitive functions leading to cognitive deficits. These cognitive deficits include impairments in learning/memory, poor decision making, and impaired control of behavioral output. Importantly, these drug-induced cognitive deficits often impact adherence to addiction treatment programs and predispose abstinent addicts to drug use relapse. Additionally, these cognitive deficits impact effective social and professional rehabilitation of abstinent addicts. The goal of this paper is to review neural substrates based on animal studies that could be pharmacologically targeted to reverse psychostimulant-induced cognitive deficits such as impulsivity and impairment in learning and memory. Further, the review will discuss neural substrates that could be used to facilitate extinction learning and thus reduce emotional and behavioral responses to drug-associated cues. Moreover, the review will discuss some non-pharmacological approaches that could be used either alone or in combination with pharmacological compounds to treat the above-mentioned cognitive deficits. Psychostimulant addiction treatment, which includes treatment for cognitive deficits, will help promote abstinence and allow for better rehabilitation and integration of abstinent individuals into society.

## Introduction

Addiction to psychostimulant drugs such as cocaine, methamphetamine, and nicotine adds a significant burden on healthcare budgets in the form of premature morbidity and mortality. Alarmingly, the use and abuse of illicit psychostimulant drugs like cocaine and methamphetamine is showing a trend of steady increase than in the last decade (1). In addition to illicit stimulant use, use and abuse of licit weak stimulant like nicotine continues to increase especially in the form of e-cigarettes and vaping (2). In addition, abuse of prescription stimulants like amphetamine, which are used to treat patients with attention deficit hyperactivity (ADHD), also adds to the problem of psychostimulant addiction. While not all people who experiment with psychostimulants will get addicted, an increasing trend of initiation does not augur well for psychostimulant addiction rates. Importantly, factors that promote transition from use/abuse to addiction are not fully understood (3, 4).

Considerable progress has been made over the last few decades in understanding the brain circuitry and pathological changes that facilitate and promote abuse of drugs (5). Despite this progress, significant challenges remain in the treatment of psychostimulant drug addiction (6). For example, currently, among the different psychostimulants described above, the Food and Drug Administration (FDA) has approved treatments for only nicotine (7, 8). Current treatment protocol for psychostimulant addiction depends largely on managing withdrawal symptoms of dependent individuals, providing behavioral/psychotherapy and utilizing self-help support groups (6). The inadequacy of current psychostimulant drug addiction is supported by high rates of relapse among abstinent addicts.

**Figure 1:**
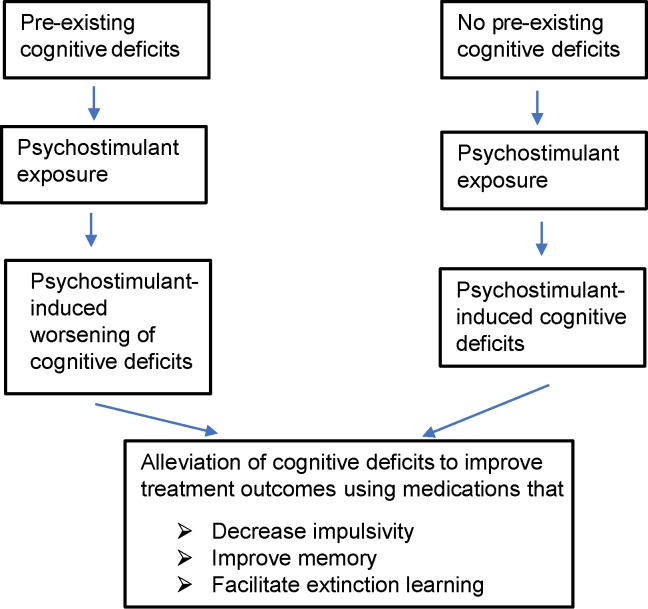
Figure shows overall hypothesis of the review and possible treatment strategies to improve outcomes of psychostimulant addiction treatment. Psychostimulant-induced cognitive deficits include impulsivity, learning/memory impairments, attentional impairment, and impairment in decision making. In this review, we mainly restrict ourselves to targets that could potentially alleviate impulsivity and/or learning/memory impairments and facilitate extinction learning. Patients with pre-existing cognitive deficits prior to drug abuse may need more aggressive treatment to break the vicious cycle of drug addiction.

The goal of behavioral/psychotherapy is to help prevent relapse among abstinent addicts by helping them develop coping strategies to deal with cravings and emotional disturbances occurring as a result of withdrawal from psychostimulant drugs (9). This requires engagement of various cognitive domains such as attention, learning, and memory. Ironically, research over the last two decades and more has demonstrated that abuse of psychostimulants results in several cognitive deficits such as impulsivity (i.e., inability to inhibit disadvantageous rapid behavioral responses), risky and/or poor decision making, impaired cognitive flexibility (i.e., impaired ability to alter behavioral responses based on changing environmental contingencies), deficits in learning and memory, and/or hyperattentiveness to drug-associated cues compared with non-drug associated cues (10–13). Interestingly, individuals with pre-existing deficits in cognition and/or suffering from psychiatric disease states that are associated with impaired cognitive function (e.g., schizophrenia and depression) are more vulnerable to abusing illicit and licit stimulants (14, 15). Importantly, recovering addicts with significant cognitive deficits are more vulnerable to relapse (12, 16). Thus, cognitive deficits in recovering drug addicts irrespective of whether they were pre-existing or drug induced need to be adequately treated to promote abstinence among drug addicts (**Figure 1**).

Among the different psychostimulant-induced cognitive deficits, this review will focus on psychostimulant-induced cognitive deficits such as impulsivity and impairments in learning and memory. The review will primarily identify neural substrates that could be pharmacologically targeted to alleviate psychostimulant-induced cognitive deficits. Finally, the review will discuss evidence from animal studies that support use of non-pharmacological approaches to alleviate the above-mentioned cognitive deficits.

## Drug-Induced Cognitive Deficits

### Impulsivity

Impulsivity in the human literature is often conceptualized as a personality trait (17). However, in the cognitive neuroscience field and for the purpose of this article, we will refer to impulsivity as behavior resulting from impaired inhibition in specific brain regions that play a role in regulating behavioral output (18). Based on the specific cognitive domains that are disrupted, impulsivity can be divided broadly into behavioral and decisional impulsivity (19). Behavioral impulsivity as the name suggests usually involves a quick behavioral response without consideration to consequences of the behavioral response (19). In contrast, decisional impulsivity involves actions and decisions taken by the individual that are less advantageous to the individual.

Psychostimulant addicts show high levels of both behavioral and decisional impulsivity than do healthy controls (20–27). It is hypothesized that this impulsive behavior is responsible for high rates of relapse among these addicts during abstinence. Consistent with these data, a recent human study reported that smokers and polysubstance abusers who abused nicotine, cocaine, and alcohol were more impulsive than controls (28).

Impulsivity observed in drug-dependent individuals can exist prior to drug abuse and worsens with repeated drug use (**Figure 1**). In support of this hypothesis, several studies have shown that individuals who are impulsive have greater sensitivity to drugs of abuse, are more likely to experiment with drugs of abuse, and are more vulnerable to develop drug dependence (29–36). This hypothesis is also supported by animal studies. For example, animals showing poor inhibitory control prior to exposure to drugs of abuse (i.e., showed more impulsive behavior) acquired cocaine self-administration behavior much more rapidly than did animals that showed good inhibitory control (37). Additionally, animals that showed more risk-taking behavior as assessed using the rodent model of Iowa gambling task (rIGT) self-administered greater amount of cocaine than did animals that did not display high risk behavior in the same task (38). However, it is not known if repeated use of drugs of abuse induces impulsivity in humans. In animals, repeated administration of cocaine, methamphetamine, and nicotine increased impulsive behavior in animals (39–43). This increase in impulsivity was observed both when animals were challenged with the drug of abuse and during withdrawal from the drug (i.e., when animals were not under the influence of the drug). Thus, these studies support the hypothesis that exposure to drugs of abuse may *de novo* induce impulsivity.

Keeping with focus of this review, we will only discuss assessment of behavioral and decisional impulsivity in animals. Behavioral impulsivity in animals can be assessed by measuring either premature responding or the ability of an animal to stop already initiated action. In animals, premature responding is measured using the five-choice serial reaction time task (5-CSRTT), while ability of an animal to stop already initiated action is measured using the stop-signal reaction time (SSRT) (44, 45) (**Box 1**). Several brain regions such as the nucleus accumbens (NAcc), dorsal striatum, infralimbic prefrontal cortex (infralimbic PFC), insula, and hippocampus have been shown to mediate behavioral impulsivity (19, 46–49). In contrast, decisional impulsivity in animals is usually assessed by measuring either temporal discounting or probability discounting. Temporal discounting is assessed using the delay discounting task (DDT), which involves assessing the ability of animal to wait for a larger reward compared with opting for an immediate smaller reward (50) (**Box 1**). Several studies have identified the role of the basolateral amygdala, orbitofrontal cortex (OFC), and hippocampus in mediating the DDT (51–53). In contrast, probability discounting is assessed using a task known as rIGT or probability discounting task and involves choosing a smaller sure reward (i.e., 100% chance to obtain the reward) compared with a larger reward, which is not always assured (i.e., approximately 50% chance or risky choice) (54, 55) (**Box 1**). Research has shown that the OFC, amygdala, habenula, and prelimbic PFC play a role in mediating probability discounting (56–58). Despite identifying the role of specific brain regions in specific types of impulsive behavior, more work is required to identify specific signaling mechanisms between the different brain regions.

Box 1 **Tasks used to measure psychostimulant-induced impulsivity.**

**Table:** 

**Task**	**Parameter measured**	**Description**
**5-Choice serial reaction time task** **(5-CSRTT)**	Behavioral impulsivity	The apparatus for the 5-CSRTT consists of five apertures. During a trial, a signal is presented in one of the apertures. Upon presentation of a signal, the animals must respond in the form of a nose poke into the aperture where the signal is presented. Nose poke in the aperture not presenting the signal is considered as an incorrect response. Similarly, response of the animal prior to presentation of a signal is considered as a premature response. Increase in premature responding is a measure of behavioral impulsivity. Lack of response by the animal is considered an omission and is indicative of impaired motor activity. Increase in incorrect responses is considered a manifestation of lack of attention. Every correct response of the animal is rewarded with a food pellet, which is collected by the animal from an aperture located on the opposite wall from the five apertures.
**Go/No-Go task**	Behavioral impulsivity	Each chamber is equipped with two retractable levers and tri-colored stimulus lights centered above each lever. The Go/No-Go trials consist of four alternating Go and No-Go components. Each component is usually 15 min long with a 5-s timeout between components for a 2-h session. During the Go component, the light on the active lever is illuminated, and a response on the active lever produces a food reward pellet on a variable interval of 30 s, and a press on the inactive lever has no consequence. Alternatively, the No-Go trial is indicated by a continuous flashing light on the active lever, and the animal must withhold a response on the active lever for a specific duration (e.g., 30 s). Responding on the active lever during the No-Go trial resets the time the animal must withhold their response (i.e., 30-s timer). The number of times the timer is reset is used as an index of behavioral impulsivity.
**Delay discounting task** **(DDT)**	Decisional impulsivity	The apparatus usually consists of three levers or apertures on one wall of the apparatus. Each lever or aperture usually has a light above it. The center aperture/lever and associated light are used to initiate trials. The two levers/apertures on either side of the center aperture are associated with rewards. Response on one of the apertures/levers is associated with immediate access to an assured small reward. In contrast, response on the other lever/aperture is associated with an assured larger reward. However, this larger reward is available after a delay. Preference of an animal for an immediate small reward compared with the delayed larger reward is suggestive of decisional impulsivity.
**Rodent version of Iowa gambling task** **(rIGT)**	Decisional impulsivity	The apparatus for the rIGT consists of five apertures like the 5-CSRTT. However, unlike the 5-CSRTT, during a trial, a signal light is presented in four apertures at the same time. Each aperture is associated with a different size of reward, and the probability of the reward is also different for each aperture. For example, responding on one of the apertures may earn the rat one pellet 90% of the time. In contrast, responding on an adjoining aperture may earn the rat four pellets, but only 40% of the time. The other two apertures may be associated with two pellets 80% or three pellets 50% of the time. Thus, the rat can choose the aperture for the amount of reward and hedge its luck. Because not all trials are rewarded, the unrewarded trials are considered punishment and are indicated by flashing light. Response of the animal prior to presentation of a signal is considered a premature response. Lack of response by the animal is considered an omission and could be indicative of impaired motor activity. Selection of aperture that is associated with larger reward but with lower probability is suggestive of risky choice and termed as “decisional impulsivity.”

#### Learning and Memory Deficits

Both learning and working memory deficits have been reported in abstinent psychostimulant addicts (21, 59–63). These learning/memory deficits are hypothesized to result in poor treatment outcomes among abstinent addicts. It is also hypothesized that working memory deficits prior to drug exposure increase vulnerability to drug addiction. Consistent with this hypothesis, individuals suffering from psychiatric disorders with significant learning and memory deficits such as depression and schizophrenia have high rates of stimulant addiction (14, 15, 64, 65). Also, a recent study reported that adolescents with weak working memory were more vulnerable to get addicted to drugs of abuse (66). In fact, acute administration of drugs like nicotine and cocaine enhances hippocampal function (67–69). Thus, individuals may compensate for memory deficits by abusing psychostimulants. Together, these findings suggest that use of psychostimulants induces memory deficits and that memory deficits present prior to drug use promote experimentation with stimulants leading to drug addiction.

Several models such as the Morris water maze, novel object recognition, and delayed match-to-sample task are used to assess learning/memory deficits in animals (**Box 2**) (70, 71). Similar to humans, chronic exposure and/or withdrawal from psychostimulants induced working memory deficits in animals. For example, animals with chronic extended-access cocaine self-administration experience showed working memory and learning deficits (72, 73). Further, animals undergoing withdrawal after chronic extended access to cocaine showed decreased functional activity of brain circuits mediating learning and memory such as the PFC, hippocampus, and striatum as measured by determining glucose utilization by these brain regions (74). Further memory deficits have been reported after withdrawal from nicotine, methamphetamine, and 3,4-methylenedioxymethamphetamine (MDMA) (75–79). Moreover, consistent with human studies, animals with memory deficits show significantly greater drug-seeking behavior than do controls. For example, neonatal ventral hippocampal lesions in rats, which lead to working memory deficits, resulted in increased reinstatement of nicotine seeking (80).

Box 2 **Tasks used to measure psychostimulant-induced memory impairment and described in this review.**

**Table:** 

**Task**	**Parameter measured**	**Description**
**Delayed match-to-sample (DMTS)**	Working memory	In this task, as the name suggests, animals are initially presented with a particular stimulus on a computer touchscreen. For example, the stimulus could be a triangle of a particular color, e.g., red. Once the animal touches the triangle, the triangle disappears from the screen. There is then predefined delay. At the end of the delay, the animal is presented with two stimuli. One of the stimuli is the previously presented red triangle. The other stimulus is new triangle of a different color, e.g., blue. Selection of the “red triangle” is considered as the correct response, while selection of the “blue triangle” is considered as the incorrect response. A high percentage of correct response is indicative of intact working memory. In contrast, a high percentage of incorrect responses is indicative of impaired working memory.
**Novel object recognition**	Episodic memory	In this task, the animal is exposed to two identical objects for a defined period of the time. The animal can explore these objects, and they are termed as familiar objects. After a period of time, which can range for 24 to 72 h, animals are again exposed to two objects. One of them is the previously exposed “familiar object,” and the other object is termed as the “novel object.” Retention of memory in the animal is determined by calculating the discrimination index, which is defined as the time spent on the novel object divided by the sum of the time spent on the novel and familiar objects. A higher discrimination index indicates intact memory. In contrast, a low discrimination index suggests impairment of memory.
**Morris water maze**	Spatial memory	The apparatus consists of black painted circular pool containing water and divided into four quadrants with four starting points. The pool contains a platform that is submerged (hidden) in the water in a particular quadrant. During training, animals are trained to locate the hidden submerged platform irrespective of the start position. During the test trials, the submerged platform is removed, and animals are placed in a quadrant opposite to the quadrant where the platform was previously hidden (quadrant of interest). The time taken for the animal to reach the quadrant of interest, the path taken to reach the quadrant of interest, and time spent in the quadrant of interest are suggestive of spatial memory. In case of impairment of spatial memory, the animal will either take longer time to reach the quadrant of interest or spend less time in the quadrant of interest or take circuitous path to reach the quadrant of interest.

In abstinent addicts, exposure to stress, drug of abuse itself, and/or drug-associated environmental cues induces cravings, which promotes drug seeking often resulting in relapse (81–84). In humans, several behavioral and cognitive therapies, such as behavioral therapy, cue exposure therapy, motivational enhancement therapy, and contingency management, are used to help abstinent addicts overcome craving (6, 85). The main goal of all these therapies is to decrease emotional and physiological responses to drug-associated cues among abstinent addicts. In animals, extinction learning is used to suppress learned responses to drug-associated cues (86–88). Extinction learning is a form of learning that involves exposure to drug-associated cues/contexts in the absence of the drug, which ultimately leads to decreased responses to drug-associated cues/contexts. In fact, reinstatement of drug seeking in response to drug-associated cues/environments after extinction training is a putative model of relapse in humans (89, 90). Several brain regions such as the infralimbic PFC, basolateral amygdala and NAcc shell, hypothalamus, and thalamus play a role in extinction learning (88). In fact, extinction learning resulted in decrease in activity of neurons in the prelimbic PFC and increase in activity of neurons in the infralimbic PFC (91–93). It has been hypothesized that facilitation of extinction learning could help in attenuating responses to drug-associated cues and prevent relapse (94, 95). Interestingly, there is significant overlap in pathways that mediate extinction of fear-associated memories and extinction of drug-associated memories (93). In fact, currently, behavioral therapies are being used to concurrently treat both substance abuse and post-traumatic stress disorder (96). Thus, in this review where direct evidence is lacking, we suggest neural substrates that play a role in extinction of fear-associated memories as possible targets for promoting extinction of drug-associated memories. It goes without saying that any such proposed targets will need to be assessed in models assessing extinction of drug-associated memories (**Box 3**). In summary, treatment of psychostimulant-dependent subjects must include procognitive agents that could alleviate working memory deficits and enhance learning/memory. Importantly, facilitation of extinction learning will help improve efficacy of cognitive behavioral therapies in humans especially cue exposure therapy.

Box 3 **Tasks used to assess facilitation of extinction learning.**

**Table:** 

**Task**	**Parameter measured**	**Description**
**Extinction of drug-induced CPP**	Extinction of drug-associated memories	In this model, a conditioned place preference (CPP) apparatus consisting of two main chambers is used. The two chambers are distinct in terms of their walls and/or floors. First, preference of the animal to the two main chambers is assessed. Next, animals are conditioned to the effects of the drug and saline/vehicle. During conditioning, animals are administered the drug and restricted to one of the chambers. Subsequently, animals are administered the vehicle/saline and restricted to the other distinct chamber. The conditioning trials are conducted either on the same day separated by at least 4–6 h or on alternate days. Once the animals are conditioned, drug-induced CPP is determined by allowing animals to assess both chambers freely. Animals will spend more time in the drug-associated chamber, suggesting rewarding effects of the drug. Subsequently, animals undergo extinction trials when they are repeatedly exposed to both chambers without drug treatment. Over a period of a few days, the time spent by the animals in the drug-associated chamber decreases, suggesting extinction of drug-induced CPP. A treatment, compared with controls, is said to facilitate extinction if the time spent by the animal in the drug-associated chamber diminishes faster.
**Extinction of drug seeking**	Extinction of drug-associated memories	In this model, animals are first trained to intravenously self-administer the concerned drug in self-administration chambers. A typical chamber has two levers—one is called the active lever and the other is called the inactive lever. Responses on the active lever are associated with drug administration. Drug administration is also associated with visual cues such as illumination of a light located above the lever. Once the animals establish stable intravenous self-administration, they undergo extinction training. During extinction training, animals can respond on either the active or inactive levers. Responses on the active lever are accompanied by neither presentation of visual cues nor drug administration. With time, responses of the animal on the active lever decrease to a point where no further decrease occurs (asymptote). A treatment is said to facilitate extinction if the animal takes fewer days to reach the lowest asymptote levels and/or if the responses on the active lever are lower compared with those of controls. In this model, reinstatement of drug seeking can be assessed by presenting the animal with drug-associated cues and by measuring responses on the active/drug-associated lever. Reinstatement of drug seeking is a putative model of relapse in humans.

## Pharmacological Targets to Treat Psychostimulant-Induced Cognitive Impairments

### Dopamine Receptors and Uptake Transporters

Changes in dopamine neurotransmission and dopamine receptors after exposure to psychostimulants like nicotine, cocaine, and methamphetamine have been previously described (97–100). Dopamine neurotransmission is primarily mediated *via* D1-like (D1 and D5) and D2-like (D2, D3, and D4) dopamine receptors. Most of the action of synaptic dopamine is terminated *via* uptake of dopamine by the dopamine uptake transporter (DAT). The dopamine uptake transporter is one of the primary targets for medications that are used to treat ADHD (101, 102). Thus, dopamine neurotransmission plays a role in both impulsivity and psychostimulant addiction. In this section, the role of D1- and D2-like dopamine receptors as possible targets for treatment of psychostimulant-induced cognitive deficits is discussed.

#### D1-Like Dopamine Receptors

Several studies have evaluated the role of D1-like dopamine receptors in impulsivity [see Jupp and Dalley (103) for review]. Blockade of D1 receptors alone after systemic administration of a D1 receptor antagonist had no influence on decisional impulsivity (104). However, blockade of D1 receptors after systemic administration of a D1 receptor antagonist in mice lacking DAT attenuated behavioral impulsivity as assessed using the 5-CSRTT (105). Interestingly, D1 receptors in specific brain regions such as the NAcc and PFC play a differential role in impulsivity. For example, blockade of D1 receptors in the NAcc core and shell decreased behavioral impulsivity (106). Consistent with these data, blockade of D1-like receptors in the NAcc shell attenuated reinstatement of cocaine seeking in rats (107). In contrast, blockade of D1-like receptors in the medial PFC (mPFC) induced decisional impulsivity (108). Together, these data suggest that D1-like receptors in specific brain regions and circuits may play a differential role in impulsivity. A recent study reported that mice lacking D1 receptors compared with control did not show premature responding after morphine exposure (109). However, the effects of D1 receptor activation and blockade in psychostimulant-induced impulsivity have not been investigated.

**Figure 2:**
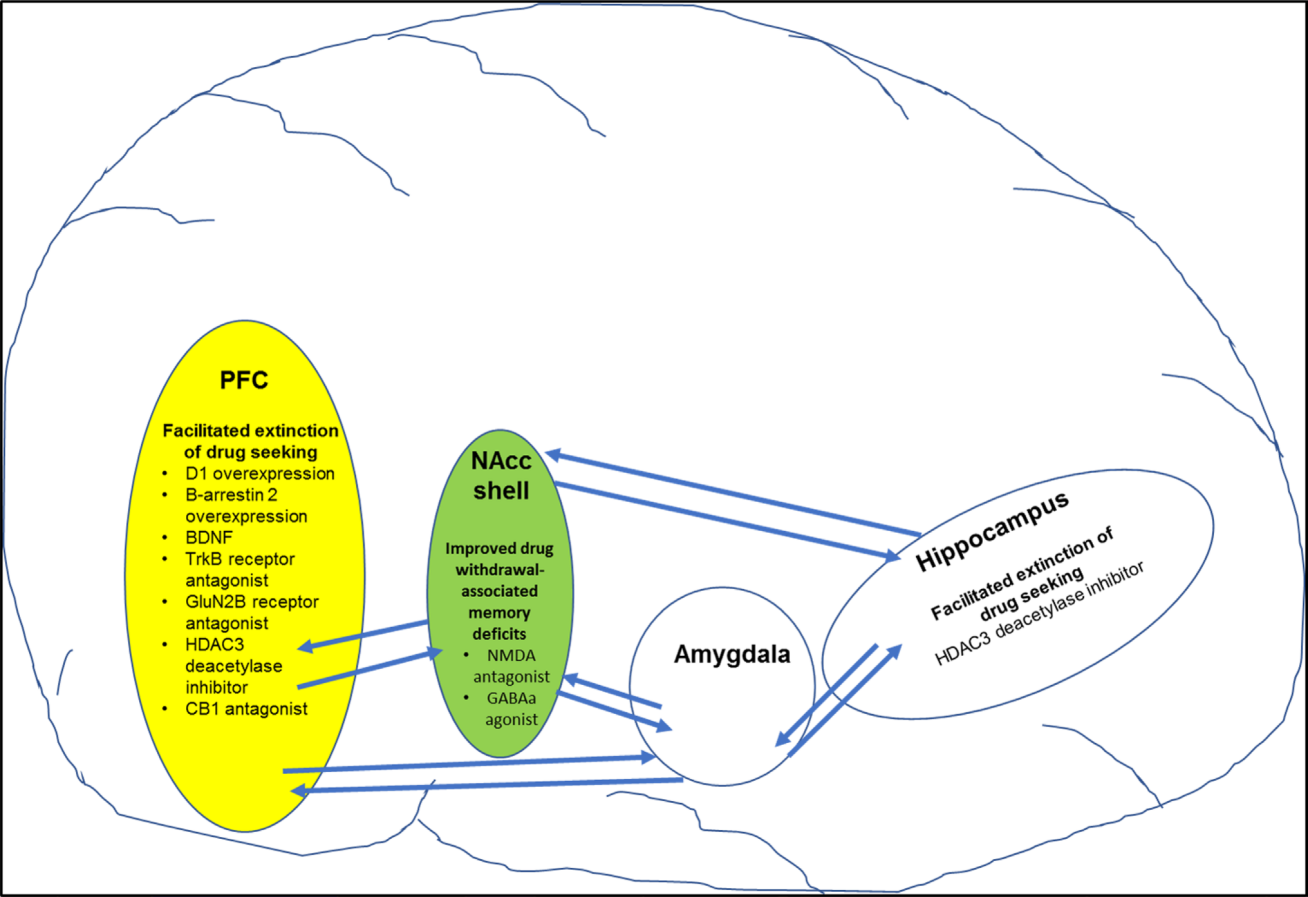
Figure shows specific targets in brain regions that play a role in improving drug-induced cognitive deficits (also see **Table 3** for more details). For example, pharmacological manipulation of targets in the prefrontal cortex (PFC) and hippocampus facilitated extinction of drug-seeking behavior. In addition, pharmacological manipulation of targets in the nucleus accumbens shell (NAcc shell) improved drug-withdrawal associated memory.

D1-mediated dopamine neurotransmission in the PFC has been shown to play a role in extinction of drug-associated memories. For example, genetically induced overexpression of D1 dopamine receptors on glutamate neurons in the PFC facilitated extinction of cocaine-induced CPP in juvenile male rats compared with controls (110) (**Table 1** ; **Figure 2**). Activation of dopamine D1-like receptors results in increase in activity of the cAMP/protein kinase A/cyclic AMP-dependent response binding element (CREB) pathway. Rolipram, a phosphodiesterase 4 (PDE-4) inhibitor, increases cAMP levels and PKA activation that resulted in facilitation of fear extinction (116). Moreover, rolipram *via* an increase in CREB levels alleviated working memory deficits associated with alcohol withdrawal (117). Withdrawal from psychostimulants is also associated with decreased activity in PKA/CREB pathway especially in brain regions mediating learning/memory such the hippocampus and PFC (118, 119). Therefore, it is possible that rolipram may help facilitate extinction learning and/or working memory deficits associated with psychostimulant withdrawal. In summary, targeting D1 receptors in specific brain regions and circuits may have utility in the treatment of psychostimulant-induced cognitive deficits especially learning and memory deficits.

**Table 1:** Brain region-specific manipulation on psychostimulant-induced cognitive deficits.

Brain region	Manipulation	Species	Task	Reward	Findings	Reference
PFC (prelimbic)	D1 receptor overexpression	Rats	Extinction of cocaine-induced CPP	Cocaine	Facilitated extinction of cocaine-induced CPP	Brenhouse et al. (110)
PFC (infralimbic)	Blockade of β receptors	Mice	Extinction of cocaine-induced CPP	Cocaine	Inhibited extinction of cocaine-induced CPP	Huang et al. (111)
PFC (infralimbic)	β-Arrestin 2 knockdown	Mice	Extinction of cocaine-induced CPP	Cocaine	Inhibited extinction of cocaine-induced CPP	Huang et al. (111)
PFC (infralimbic)	β-Arrestin 2 overexpression	Mice	Extinction of cocaine-induced CPP	Cocaine	Facilitated extinction of cocaine-induced CPP	Huang et al. (111)
PFC (infralimbic)	BDNF	Rats	Extinction of cocaine-induced CPP	Cocaine	Facilitated extinction of cocaine-induced CPP	Otis et al. (112)
PFC (infralimbic)	TrkB receptor antagonist (ANA-12)	Rats	Extinction of cocaine-induced CPP	Cocaine	Inhibited extinction of cocaine-induced CPP	Otis et al. (112)
PFC (infralimbic)	GluN2B receptor antagonist ifenprodil	Rats	Extinction of cocaine-induced CPP	Cocaine	Inhibited extinction of cocaine-induced CPP	Otis et al. (112)
PFC (infralimbic)	HDAC3 deacetylase inhibitor	Rats	Extinction of cocaine-induced CPP	Cocaine	No effect on extinction of cocaine-induced CPP	Alaghband et al. (113)
PFC	CB1 antagonist (rimonabant)	Mice	Extinction of cocaine-induced CPP	Cocaine	Facilitated extinction of cocaine-induced CPP	Hu et al. (114)
NAcc shell	GABA_A_ agonist (muscimol)	Rats	Morris water maze	Methamphetamine	Improved methamphetamine withdrawal induced spatial memory deficit	Heysieattalab et al. (115)
NAcc shell	GABA_A_ antagonist (bicuculline)	Rats	Morris water maze	Methamphetamine	Worsened methamphetamine withdrawal induced spatial memory deficit	Heysieattalab et al. (115)
NAcc shell	NMDA antagonist (AP-5)	Rats	Morris water maze	Methamphetamine	Improved methamphetamine withdrawal induced spatial memory deficit	Heysieattalab et al. (115)
Dorsal hippocampus	HDAC3 deacetylase inhibitor	Rats	Extinction of cocaine-induced CPP	Cocaine	Facilitated extinction of cocaine-induced CPP	Alaghband et al. (113)

BDNF, brain-derived neurotrophic factor; Trk B, tropomyosin-related kinase B.

#### D2-Like Dopamine Receptors

Acute cocaine dose dependently decreased decisional impulsivity in rats as assessed using the DDT (120). The same study showed that systemic administration of D2 receptor antagonist, eticlopride, reversed acute cocaine-induced inhibition of decisional impulsivity, suggesting that the effects of cocaine on decisional impulsivity are mediated by D2 receptor activation. Further, the study showed that D2 receptors in the amygdala possibly mediate the inhibitory effect of acute cocaine on decisional impulsivity. Chronic cocaine exposure decreased striatal D2 receptor mRNA in both high and low impulsive rats and selectively decreased immediate early gene zif268 mRNA in the OFC and infralimbic cortices of high impulsive animals (121). Thus, impulsive behavior observed after chronic cocaine exposure was possibly due to decreased D2-mediated dopamine signaling in the above-described brain regions.

D2 dopamine receptors located in the NAcc, ventral tegmental area (VTA), and PFC also play a role in impulsive behavior. Specifically, D2/3 receptor availability was significantly decreased in the NAcc of high impulsive rats compared with low impulsive rats (122, 123). Further chronic methylphenidate treatment decreased impulsivity in high impulsive rats by increasing expression of D2 receptor availability in the dorsal striatum and NAcc (123). Similarly, decreased D2-mediated dopamine transmission in the PFC and VTA induced decisional impulsivity (124, 125). Interestingly, systemic administration of D2 agonist ropinirole induced decisional impulsivity as assessed using the rIGT (126). However, ex vivo analyses of brain slices revealed that chronic ropinirole treatment led to upregulation of the β-arrestin-AKT-GSK3β intracellular cascade, which usually suggests D2-mediated signaling under hyperdopaminergic conditions.

Interestingly, activation of D3 receptors induced decisional impulsivity as assessed using the rIGT (109, 127–129). In contrast, blockade of D3 receptors decreased decisional impulsivity. In addition, blockade of D3 receptors attenuated cocaine and methamphetamine seeking (130, 131). Together, the data suggest that blockade of D3 receptors may help to attenuate decisional impulsivity and drug seeking. Further studies are required to assess the effects of D3 antagonists on psychostimulant-induced impulsivity.

In summary, the above-described evidence suggests that D2-mediated dopamine neurotransmission in specific brain regions such as striatum and mPFC receptors may help to alleviate decisional impulsivity associated with psychostimulant addiction (**Box 4**). In contrast to D2 receptors, blockade of D3 dopamine receptors may help alleviate psychostimulant-induced decisional impulsivity. Overall, D2-like dopamine receptors are useful targets in the treatment of psychostimulant addiction.

Box 4 **Potential targets/approaches for alleviation of psychostimulant-induced impulsivity, memory impairment, and/or facilitation extinction of drug-associated memories. BDNF, brain-derived neurotrophic factor; CRF, corticotrophin-related factor; Trk B, tropomyosin-related kinase B; nACh, nicotinic acetylcholine; IGF, insulin growth factor; PAM, positive allosteric modulator; PPARγ, peroxisome proliferator agonist receptor gamma.**

**Table:** 

**Decreasing impulsivity**	**Reversing memory impairment**	**Facilitating extinction learning**
α2 agonists	NMDA receptor antagonists	mGlu5 receptor PAM
NET blockers	mGlu5 receptor PAM	AMPA receptor agonist
Orexin receptor antagonists	*N*-Acetylcysteine, riluzole	Glycine receptor coagonist
CB1 receptor antagonists	α7 nACh receptor agonist/PAM	GABA_B_ agonist
D3 receptor antagonists	CB1 antagonists	Phosphodiesterase inhibitors
α4β2 nACh receptor antagonists	Activation of PKCε	Orexin receptor antagonists
mGlu4 PAM	Insulin	Increase BDNF levels
mGlu2/3 agonists	PPARγ agonists	TrK B receptor activation
5-HT3, 5-HT2A, and 5-HT2c antagonists	IGF-2 agonists	Increase in oxytocin levels
5-HT1A agonist	Exercise	Increase in ghrelin levels
Progesterone	Brain stimulation	CRF receptor antagonists
Exercise	Neurogenesis	IGF-2 agonists
		17β-estradiol
		Exercise
		Brain stimulation

### Adrenergic Receptors and Noradrenergic Reuptake Transporters

The role of noradrenaline in impulsivity is evident by use of medications that increase noradrenergic transmission in the treatment of ADHD (101, 102). Noradrenergic transmission is mediated by α (α1 and α2) and β (β1 and β2) adrenergic receptors, and the action of synaptic noradrenaline is terminated by the noradrenaline uptake transporter (NET). Several drugs approved by the FDA for ADHD treatment include α2 adrenergic receptor agonists (e.g., guanfacine and clonidine), NET and DAT inhibitors (e.g., amphetamine and methylphenidate), and selective NET inhibitor (e.g., atomoxetine). Importantly, exposure to psychostimulants like cocaine, nicotine, and methamphetamine alters noradrenergic neurotransmission in the brain (132–135). In this section the role of α2, β2, and NET in psychostimulant-induced cognitive deficits is discussed.

#### **α**2 Adrenergic Receptors and NET

Like in humans, drugs that increase noradrenergic transmission decreased impulsivity in animal models (136–138). Guanfacine, a selective α2A adrenergic receptor agonist, attenuated cocaine-induced behavioral impulsivity and memory impairment in monkeys (139) (**Tables 2** and **3**). More recently, it was reported that guanfacine improved inhibitory control in abstinent cocaine-dependent subjects (153). Also, α1 and α2 adrenergic receptor agonists decreased reinstatement of cocaine seeking (154). In contrast, α2 adrenergic receptor antagonist yohimbine is commonly used to pharmacologically induce reinstatement of psychostimulant drug seeking (155).

**Table 2:** Pharmacological alleviation of psychostimulant-induced impulsivity in animals.

Target	Task	Species	Drug	Findings	Reference
α2A adrenergic receptor agonists (guanfacine)	5-CSRTT	Rats	Acute cocaine	Dose-dependent decrease in behavioral impulsivity	Terry et al. (139)
Orexin receptor antagonist (suvorexant)	5-CSRTT	Rats	Acute cocaine	Decreased behavioral impulsivity but had no effect on decisional impulsivity	Gentile et al. (140)
Progesterone	Go/No-Go task	Rats	Acute cocaine	Decreased behavioral impulsivity in female but not male rats	Swalve et al. (141)
NET uptake blocker (atomoxetine)	DDT	Rats	Acute cocaine	Decreased decisional impulsivity in male rats compared with controls; no effect of atomoxetine alone in females	Smethelss et al. (142)
CB1 antagonists (rimonabant)	DDT	Rats	Repeated cocaine exposure	Both prevented and reversed cocaine-induced decisional impulsivity	Hernandez et al. (39)

5-CSRTT, 5-choice serial reaction time task; DDT, delay discounting task.

**Table 3:** Pharmacological alleviation of psychostimulant-induced memory impairment and/or facilitation of extinction learning.

Compounds	Task	Species	Drug treatment	Findings	Reference
α2A adrenergic receptor agonists (guanfacine)	Delayed match-to-sample (DMTS)	Monkeys	Acute cocaine	Alleviated cocaine-induced impairment in accuracy in the DMTS task suggesting improvement in working memory	Terry et al. (140)
NMDA antagonist (memantine)	Novel object recognition	Rats	Amphetamine withdrawal	Attenuated amphetamine withdrawal-induced impairment in memory	Marszalek-Grabska et al. (145)
CB1 antagonist (rimonabant)	Novel object recognition	Mice	Nicotine withdrawal	Attenuated nicotine withdrawal-induced impairment in memory	Saravia et al. (146)
Glycine site partial agonist (d-cycloserine)	Extinction of cocaine-induced CPP	Rats	Cocaine	Facilitated extinction of cocaine-induced CPP	Botreau et al. (147)
mGlu5 PAM (CDPPB)	Extinction of cocaine-induced CPP	Rats	Cocaine	Facilitated extinction of cocaine-induced CPP	Gas and Olive (148)
	Extinction of cocaine seeking	Rats	Cocaine	Facilitated extinction of cocaine seeking	Cleva et al. (149)
PD4 inhibitor (rolipram)	Extinction of cocaine-induced CPP	Mice	Cocaine	Facilitated extinction of cocaine-induced CPP	Liddie et al. (150)
PD9 inhibitor (BAY-73-6691)	Extinction of cocaine-induced CPP	Mice	Cocaine	Facilitated extinction of cocaine-induced CPP	Liddie et al. (150)
TrK B agonist	Extinction of cocaine-induced CPP	Rats	Cocaine	Facilitated extinction of cocaine-induced CPP	Otis at al. (112, 113)
17β estradiol	Extinction of cocaine-induced CPP	Rats	Cocaine	Facilitated extinction of cocaine-induced CPP	Twining et al. (151)
Vagal nerve stimulation	Extinction of cocaine seeking	Rats	Cocaine	Facilitated extinction of cocaine seeking	Childs et al. (152)
GABA_B_ agonist (baclofen)	Extinction of methamphetamine-induced CPP	Rats	Methamphetamine	Facilitated extinction of methamphetamine-induced CPP	Voigt et al. (153)

DMTS, delayed matching to sample task; CPP, conditioned place preference.

In addition, direct injection of atomoxetine in the NAcc shell, but not NAcc core or the PFC, reduced behavioral impulsivity as assessed using the 5-CSRTT (156). However, decisional impulsivity as measured using DDT was not altered by atomoxetine injections into either the mPFC or OFC (125). Importantly, relevant to this review, atomoxetine reduced decisional impulsivity for cocaine rewards using the DDT in male rats [(144) (**Table 2**), but see Ref. (157)]. In contrast, atomoxetine alone did not attenuate decisional impulsivity associated with cocaine rewards in female rats. However, decisional impulsivity for cocaine rewards in females was attenuated after treatment with either progesterone alone or progesterone in combination with atomoxetine. Together, the data suggest atomoxetine may not be as effective in female compared with male cocaine abusers, suggesting a role for gender in psychostimulant-induced impulsivity treatment (discussed later). Systemic administration of atomoxetine also attenuated reinstatement of cocaine seeking (157–159). Overall, the data support the role of α2 adrenergic receptor agonists and/or selective NET inhibitors in the treatment of psychostimulant-induced impulsivity (**Box 4**).

#### **β** Adrenergic Receptors

The role of noradrenergic neurotransmission *via* β adrenergic receptors has been explored in both consolidation of drug-associated memory and extinction learning. Specifically, administration of β receptor antagonist propranolol immediately after nicotine administration attenuated reinstatement of nicotine seeking in animals (160). Consistent with these findings, administration of propranolol attenuated craving among abstinent smokers for a novel conditioned stimulus associated with nicotine. Inhibition of hippocampal β receptors attenuated expression of cocaine-associated memory as assessed using the cocaine-induced CPP model (113). Importantly, propranolol facilitated extinction of fear in rabbits (162). However, a recent study has reported that direct injections of propranolol in the infralimbic PFC attenuated extinction learning of cocaine-induced CPP *via* inhibition of ERK-signaling pathway (111) (**Table 1**; **Figure 2**). In fact, the study also reported that overexpression of β-arrestin 2 in the infralimbic PFC promoted extinction of cocaine-induced CPP. Further, knockout of β-arrestin 2 in the infralimbic PFC impaired extinction of cocaine-induced CPP. Taken together, the data suggest a role for β-adrenergic receptors in facilitating extinction of drug-associated memories. Further, development of β-adrenergic ligands that selectively promote signaling *via* β-arrestin 2 pathway will help in better understanding the role of β-adrenergic receptors in extinction learning. In summary, α2 and β adrenergic receptors and NET are very viable targets for treatment of cognitive impairments associated with psychostimulant addiction. Future work must focus on determining specific circuits that are targeted by α2 and β adrenergic receptor agonists and/or selective NET inhibitors to decrease impulsivity and facilitate working memory and/or extinction of drug-associated cues.

### Serotoninergic Receptors

Alteration in serotoninergic neurotransmission after chronic exposure to cocaine and other psychostimulants has been previously described (163). Serotoninergic tone in the brain plays an important role in inhibitory control (164). Several lines of evidence suggest that a decrease in brain serotonin (5-HT) increases impulsivity, while elevation of brain 5-HT levels decreased impulsivity (165–167). Interestingly, increased 5-HT release in the PFC was found to be associated with higher levels of behavioral impulsivity as assessed using the 5-CSRTT (168, 169). Overall, a majority of the data suggest that elevation of serotoninergic transmission improves impulsive behavior.

In addition to 5-HT, several studies support a role of both 5-HT2A and 5-HT2C receptors in impulsive behavior. For example, 5-HT2A receptor expression in the mPFC was higher in high compared with low impulsive animals (170). Further, activation of 5-HT2A receptors induced behavioral impulsivity, while blockade of 5-HT2A receptors inhibited behavioral impulsivity (170, 171). Moreover, 5-HT2A receptor activation in the OFC induced decisional impulsivity (172). Future work needs to assess the effects of 5-HT2A receptor antagonists in psychostimulant-induced impulsivity. Similar to 5-HT2A, 5-HT2C receptor expression was significantly greater in the OFC in high compared with low impulsive animals (122). In contrast, no difference in 5-HT2C receptor expression was reported in the striatum between high and low impulsive animals. Blockade of 5-HT2C receptors selectively improved decisional impulsivity in the rIGT (173). Chronic cocaine self-administration decreased 5-HT2C receptor expression in the NAcc shell in the high impulsive animals but decreased 5-HT2C receptor expression in the infralimbic PFC in the low impulsive animals (122). Together, the data suggest that cocaine differentially influences 5-HT2C receptor expression in different brain regions depending on the impulsivity in the animals prior to cocaine exposure. Based on the above data, it is hypothesized that 5-HT2C receptor antagonists will attenuate psychostimulant-induced impulsivity.

5-HT3 antagonists, granisetron and ondansetron, decreased decisional impulsivity in the DDT (173) (**Box 4**). This decrease in decisional impulsivity was not observed after administration of the 5-HT reuptake blocker (paroxetine) or the 5-HT1A receptor agonist (8-OH-DPAT). Interestingly, infusion of 5-HT1A receptor agonist 8-OH-DPAT into the OFC decreased decisional impulsivity (124). Together, 5-HT3 receptor antagonists and 5-HT1A receptor agonists could be potentially useful in treating psychostimulant-induced impulsivity. In summary, establishing serotoninergic tone in psychostimulant-dependent subjects may help ameliorate cognitive deficits induced by abuse of psychostimulants. Further, 5-HT1A, 5-HT2A, 5-HT2C, and 5-HT3 receptors are possible targets that need to be further explored for the treatment of psychostimulant-induced impulsivity.

### GABA Receptors

Exposure to psychostimulants like cocaine, nicotine, and methamphetamine alters GABAergic neurotransmission (174–176). GABA also plays a role in both impulsivity and learning/memory (177–179). Activation of GABA_A_ receptors in the mPFC induced behavioral impulsivity, while blockade of GABA_A_ receptors in the same region reduced behavioral impulsivity (180–182). Moreover, activation of GABA_A_ receptors in the lateral habenula increased cue-induced cocaine seeking, suggesting lack of inhibitory control in response to drug-associated cues (183). Importantly, activation of GABA_A_ receptors in the NAcc shell improved methamphetamine-induced working memory deficit as measured using the Morris water maze (115). Together, the data suggest that GABA_A_-mediated neurotransmission in different brain regions plays a differential role in psychostimulant-induced impulsivity and memory deficits (**Box 4**).

In addition to GABA_A_ receptors, GABA_B_ receptors play a role in drug seeking. For example, GABA_B_ agonists and positive allosteric modulators (PAMs) attenuated reinstatement of nicotine and cocaine seeking (174, 184, 185). Importantly, activation of GABA_B_ receptors facilitated extinction of methamphetamine-induced CPP (152) (**Table 3**). In summary, both GABA_A_ and GABA_B_ receptors mediate psychostimulant-induced cognitive deficits. However, further work is required to fully exploit the potential of GABA_A_ and GABA_B_ receptors in the treatment of psychostimulant-induced cognitive deficits.

### Glutamate Neurotransmission

Dysregulation in glutamate transmission has been reported after exposure to psychostimulants (174, 186–188). Further, research has shown that both ionotropic and metabotropic glutamate (mGlu) receptors play a role in impulsivity, memory deficits, and extinction of drug-associated memories (189, 190) (**Box 4**).

#### NMDA Receptors

Systemically administered GluN2B antagonists Ro 63-1908 and traxoprodil increased premature responses in the 5-CSRTT, suggesting behavioral impulsivity (191). Similarly, systemic administration of NMDA antagonists induced decisional impulsivity as assessed using the DDT [(192, 193); but also see Higgins et al. (191)]. Together, the data suggest that blockade of NMDA-mediated glutamate transmission after systemic administration of NMDA antagonists induced behavioral and decisional impulsivity. However, blockade of NMDA receptors in specific brain regions had a differential effect on behavioral and decisional impulsivity. For example, blockade of NMDA receptors in the infralimbic PFC induced behavioral impulsivity (181). In contrast, blockade of GluN2B-containing NMDA receptors in the NAcc core induced decisional impulsivity in rats (194). Together, these data suggest that NMDA-mediated glutamate transmission in the infralimbic PFC and NAcc core plays a role in behavioral and decisional impulsivity, respectively. The effects of NMDA-antagonists and specifically GluN2B antagonists in psychostimulant-induced impulsivity still need to be assessed.

The role of NMDA-mediated glutamate transmission in learning and memory including extinction learning is well documented (189, 195, 196). Relevant to this review, systemic administration of NMDA antagonist memantine improved amphetamine withdrawal-induced memory deficit as assessed using the novel object recognition test (143) (**Table 3**). Similarly, blockade of NMDA receptors in the NAcc using NMDA antagonist AP-5 improved methamphetamine-induced working memory deficit as measured using the Morris water maze (115) (**Table 1**; **Figure 2**). Interestingly, increasing activity of NMDA-mediated glutamate transmission *via* manipulation of the glycine site facilitated extinction of fear- and cocaine-associated memories (145, 197, 198) (**Table 3**). In addition, increased NMDA-mediated transmission especially *via* GluN2B-containing NMDA receptors facilitated extinction of fear memories (199). Consistent with these findings, increasing glutamate transmission *via* GluN2B-containing NMDA receptors in the infralimbic cortex facilitated extinction of cocaine-associated memory (112) (**Table 1**; **Figure 2**). More recently, it was reported that aquaporin-4 (AQP-4) deletion increased GluN2B-mediated glutamate transmission in the CA3–CA1 hippocampal pathway (200). AQP-4 is the predominant water channel primarily expressed in astrocytes and plays a role in regulating synaptic plasticity. Importantly, deficiency of AQP-4 facilitated fear memory extinction (200). Future work must investigate if AQP-4 may be a potential target for facilitating extinction of psychostimulant drug-associated memories. In summary, NMDA receptors could serve as targets for alleviation of psychostimulant-induced impulsivity and memory deficits. Furthermore, NMDA receptors could be targeted to facilitate extinction of drug-associated memories.

#### AMPA Receptors

The AMPA receptors are also involved in extinction learning. For example, AMPA receptor activation facilitated extinction of fear-associated memories (199, 201). Activation of AMPA receptors in the infralimbic PFC facilitated extinction of heroin-associated memories (202). However, the effects of AMPA receptor activation on extinction of psychostimulant-associated memories have not been evaluated. Surface expression of AMPA receptors can be regulated by a process called ubiquitination. Ubiquitination of AMPA receptors results in internalization of AMPA receptors, which indirectly decreases AMPA-mediated glutamate transmission. More recent work has shown that ubiquitination of AMPA receptors is partially regulated by epidermal growth factor receptor substrate 15 (Eps15). Decreased expression of Eps15 resulted in decreased internalization of GluA1-containing AMPA receptors possibly by decreased ubiquitination of GluA1 subunits of the AMPA receptors (204). However, further work is required to determine if knockdown of Eps15 facilitates extinction learning *via* decreased internalization of AMPA receptors. In summary, Eps15 *via* AMPA-mediated glutamate transmission could be a potential target to facilitate extinction learning of psychostimulant-associated memories.

#### Metabotropic Glutamate (mGlu) Receptors

Several experimental studies support the role of mGlu receptors in cognitive deficits. For example, blockade of mGlu1 receptors resulted in decisional impulsivity (195). These data suggest that PAMs of the mGlu1 receptors may help to reduce decisional impulsivity, although this hypothesis needs to be experimentally tested in psychostimulant-induced impulsivity. In addition to mGlu1 receptors, mGlu2/3 receptors also mediate behavioral and decisional impulsivity. Pretreatment with the mGlu2/3 agonist LY379268 attenuated 5-HT2A agonist-induced behavioral impulsivity (205). Furthermore, direct injections of mGlu2/3 agonist in the OFC and mPFC attenuated intra-OFC and intra-PFC 5-HT2A agonist-induced decisional and behavioral impulsivity, respectively (172). Moreover, systemic administration of mGlu4 PAM, 4-((*F*)-styryl)-pyrimidin-2-ylamine (Cpd11), induced behavioral impulsivity but decreased decisional impulsivity (206). In contrast to mGlu4 receptors, blockade of mGlu5 receptors, using a mGlu5 negative allosteric modulator (NAM), attenuated behavioral impulsivity (207). In addition, activation of mGlu5 receptors, using a mGlu5 receptor PAM, attenuated NMDA antagonist MK-801-induced behavioral impulsivity. Interestingly, no effects of mGlu5 receptor modulation on decisional impulsivity were observed (207). Importantly, systemic administration of mGlu5 PAM, CDPPB, facilitated extinction of fear- and cocaine-associated memories (148, 149, 208) (**Table 3**). Consistent with these findings, decreased glutamate transmission *via* mGlu1 and mGlu5 receptors in the mPFC facilitated resistance to extinction of cocaine-associated memories in animals with chronic cocaine self-administration experience (209). Taken together, the data suggest that mGlu5 receptors have a role in behavioral impulsivity and can also be targeted to facilitate extinction of psychostimulant-associated memories. Based on the above-described data, mGlu1, mGlu2/3, mGlu4, and mGlu5 receptors can serve as potential targets in psychostimulant-induced impulsivity.

#### Drugs That Restore Glutamatergic Tone

As described above, dysregulation in glutamate transmission has been reported after exposure to psychostimulants. Thus, agents that restore glutamatergic tone may help to treat psychostimulant addiction. Administration of riluzole, a pharmacological compound that reestablishes glutamatergic tone, decreased activity of the prelimbic PFC and increased activity of the infralimbic PFC (210). Also, direct injections of riluzole in the amygdala facilitated extinction of fear-associated memories, possibly due to the increase infralimbic PFC activity (211). Importantly, riluzole attenuated reinstatement of cocaine seeking (210). Like riluzole, *N*-acetylcysteine, a cystine–glutamate antiporter that helps restore glutamatergic tone, attenuated reinstatement of cocaine and nicotine seeking (212, 213). *N*-Acetylcysteine also reduced reinstatement of nicotine seeking observed in animals with neonatal ventral hippocampal lesions (80, 214). As described above, animals with neonatal ventral hippocampal lesions show memory deficits and higher nicotine seeking than do controls. It is hypothesized that memory deficits associated with ventral hippocampal lesions are partially responsible for this increased nicotine seeking. Together, the data suggest that *N*-acetylcysteine helps animals overcome memory deficits and thus possibly helps reduce drug seeking. Overall, the above data with riluzole and *N*-acetylcysteine suggest that correcting the dysregulation in glutamate transmission can improve memory deficits and/or facilitate extinction learning. Future work needs to determine if these drugs can facilitate extinction of drug-associated memories. In summary, both ionotropic and metabotropic glutamate receptors are viable targets for treatment of psychostimulant-induced cognitive deficits. However, more work is required to understand glutamate dysregulation in specific brain circuits after psychostimulant exposure to fully exploit the various glutamate targets for treatment of psychostimulant-induced cognitive deficits.

### Nicotinic Acetylcholine Receptors (nAChRs)

The role of nAChRs in impulsive behavior has been discussed previously (215). In humans, polymorphism in the α4 subunits of the nAChRs (CHRNA4) was associated with pathological gambling in Korean gamblers (216). Also, systemic administration of varenicline, a partial agonist of α4β2 nAChRs, induced behavioral impulsivity in animals (217). Importantly, blockade of α4β2 nAChRs in the infralimbic PFC attenuated varenicline-induced behavioral impulsivity, suggesting that the effects of varenicline are mediated by α4β2 nAChRs in the infralimbic PFC (218). Also, intra-cerebroventricular injection of α4β2 nAChR antagonist decreased behavioral impulsivity in animals (219). Together, the data highlight that the role of α4β2 nAChRs in behavioral impulsivity and α4β2 nAChR antagonists may help to alleviate behavioral impulsivity. More recently, compounds that decrease signaling *via* α4β2 nAChRs attenuated cocaine and methamphetamine seeking (220). In addition, systemic administration of partial agonists of the α7-containing nAChRs decreased behavioral impulsivity and improved attention as assessed using the five choice-continuous performance task (5-CCPT) (221). The decrease was specifically observed in female rats that had been classified as animals with low attention at baseline. α7 nAChR agonists have also been shown to improve memory (222, 223). Together, the data suggest a possible role for α4- and α7-containing nAChRs in cognitive deficits such as impulsivity and impairment of memory. However, the role of the different nAChR subunits in psychostimulant-induced cognitive deficits is yet to be evaluated.

### Opioid Receptors

Several lines of evidence support the role of endogenous opioids in impulsive behavior. For example, human imaging studies suggest upregulation of µ opioid receptor (MORs) in the mPFC and OFC in individuals with traits suggestive of impulsivity (223). Further, pathological gamblers, who are known to be impulsive and impaired in making rational decisions, show decreased endogenous opioid release in the brain than do healthy volunteers (224). Consistent with these findings, administration of MOR antagonist decreased pathological gambling (225, 226). MORs and opioid peptides are extensively found in PFC and regulate PFC neuronal activity (227). Activation of MORs in the PFC induced behavioral impulsivity (228). In addition, mice lacking MORs showed markedly decreased behavioral impulsivity (229). In contrast, the same study showed that mice lacking delta opioid receptors (DORs) showed increased behavioral impulsivity. More recently, it was reported that α2 agonist yohimbine-induced increase in behavioral impulsivity was attenuated by blockade of kappa opioid receptors (KORs) (230). Interestingly, KOR activation on its own decreased behavioral impulsivity possibly due to impairment of motor activity. Together, the data from these pharmacological and genetic studies suggest a differential role for MORs, DORs, and KORs in behavioral impulsivity. Further, the data suggest that MOR and KOR antagonists may help to reduce impulsivity.

Chronic cocaine administration is associated with upregulation of MORs and KORs in the PFC (231). Furthermore, upregulation of MORs in the anterior cingulate cortex predicts both severity of craving and relapse in cocaine users (232, 233). Similarly, dysregulation of endogenous opioid neurotransmission occurs after exposure to nicotine (234). However, much work needs to be done in determining the role of MORs, MOR antagonists, and other opioid receptors in psychostimulant-induced cognitive deficits especially impulsivity (**Box 4**). Moreover, most of the research on the role of opioid receptors in impulsivity has focused on the PFC. However, further work must be carried out in other brain regions to determine the role of endogenous opioids in psychostimulant-induced impulsivity.

### Cannabinoid Receptors

The endogenous cannabinoid system is altered after exposure to psychostimulant drugs. For example, exposure to cocaine administration during adolescence increased expression of CB1 receptors and decreased expression of CB2 receptors in the PFC and hippocampus (235). In contrast in adult rats, chronic cocaine self-administration resulted in decreased CB1 and CB2 receptor expression in the PFC, dorsal striatum, and amygdala (236). Further, blockade of CB1 receptors attenuated both cocaine intake and reinstatement of cocaine seeking (237). Importantly, blockade of CB1 receptors prevented cocaine-induced impairment in decisional impulsivity as assessed using the DDT (39) (**Table 2**). In addition, acute administration of CB1 antagonists prior to DDT in cocaine-treated rats reversed cocaine-induced decisional impulsivity. Together, these data support a role for endogenous cannabinoids in both preventing and reversing cocaine-induced impulsivity. Consistent with the findings of this study, activation of CB1 receptors using cannabidiol (CBD) did not improve impulsivity during tobacco abstinence in human smokers (238).

In addition, CB1 receptors located in the amygdala and hippocampus play a role in learning and memory. For example, blockade of CB1 receptors attenuated nicotine withdrawal-induced memory deficits (144) (**Table 3**). Interestingly, the same study also showed that selective deletion of CB1 receptors in the GABA neurons also mitigated nicotine withdrawal-induced memory deficits. Overall, these data suggest that CB1 receptor antagonists may have therapeutic utility in promoting smoking cessation by decreasing memory deficits associated with nicotine withdrawal. Importantly, systemic or intra-mPFC administration of CB1 receptor antagonist rimonabant enhanced extinction of cocaine-associated memories (114) (**Table 1**; **Figure 2**). Together, these data suggest that CB1 receptor antagonists could be potentially used to treat psychostimulant-induced impulsivity and memory impairment (**Box 4**). However, further research is required to fully exploit the potential of the endocannabinoid system as a potential treatment for psychostimulant-induced cognitive deficits.

### Phosphodiesterase Inhibitors

The role of cAMP/protein kinase A/cyclic-AMP response element binding (CREB) protein pathway plays an important role in both memory and reinforcing effects of psychostimulant drugs (239–241). The enzyme phosphodiesterase (PDE) plays a role in breakdown of cAMP and thus indirectly decreases CREB formation. Phosphodiesterase inhibitors, which increase CREB formation, facilitate learning and memory (242). For example, subchronic administration of rolipram, a PDE4 inhibitor, using osmotic pumps facilitated learning of conditioned fear (243). Importantly, more recently it has been reported that rolipram facilitated extinction of fear-associated memory in mice (116). Interestingly, PDE4 inhibitors did not facilitate extinction of cocaine-induced CPP (148) (**Table 3**). However, the same study showed that PDE9 inhibitor BAY-73-6691 facilitated extinction of cocaine-induced CPP (**Table 3**). This effect of PDE9 inhibitor was possibly mediated by an increase in cGMP levels in the hippocampus and amygdala. Further work is required to assess the effects of PDE9 inhibitors and other PDE inhibitors in facilitation of extinction of drug-associated memories. In summary, the various isoforms of the PDE enzyme continue to be viable targets for treatment of psychostimulant addiction.

### Orexin

Orexin neurons (also referred to as hypocretin) are found in the hypothalamus and release the neuropeptides orexin A and orexin B (also referred to as hypocretins 1 and 2) throughout the CNS (244). With its widespread targets, the orexin system is involved in a number of functions including stress, reward, wakefulness, and food seeking (245). The hypocretin/orexin system plays an important role in the reinforcing effects of cocaine. For example, suvorexant, a dual orexin receptor antagonist, attenuated both the rewarding and motivational effects of cocaine (141). Also, knockdown of hypocretin/orexin neurons in the dorsal hypothalamus attenuated cocaine self-administration (246). Further knockdown of orexin 1 receptor in the VTA both altered dopamine signaling in the NAcc and attenuated cocaine-induced increase in NAcc DA (248). The hypocretin/orexin system also plays a role in opioid- and alcohol-dependent behaviors (249, 250).

Increased activation of medial hypothalamic orexin neurons, but not lateral hypothalamic neurons, was reported during a Go/No-Go task involving food reward, suggesting a role for medial hypothalamic orexin neurons in behavioral impulsivity (251). More recently, systemic or intra-VTA administration of suvorexant, a dual orexin receptor antagonist, attenuated cocaine-induced behavioral impulsivity (140) (**Table 2**). Interestingly, neither suvorexant nor orexin 1 (SB334867) nor orexin 2 (TCS-OX2-29) receptor-selective compounds altered decisional impulsivity. Taken together, the data suggest that orexin receptor antagonists may be useful in reducing psychostimulant-induced behavioral impulsivity.

The hypocretin/orexin receptors are also found in brain regions that play a role in memory especially the hippocampus. Administration of orexin peptides increased firing of hippocampal neurons and facilitated learning and memory (252–256). The orexin-induced facilitation of learning is mediated by increasing neurogenesis in the hippocampus (257). The hypocretin/orexin system also plays a role in extinction learning. For example, blockade of orexin 1 receptor facilitated extinction of fear-associated memories possibly by increasing amygdalar input to the infralimbic PFC during extinction learning (258). However, the role of the orexin system in facilitation of extinction of drug-associated memories has not been explored. In summary, blocking orexin-mediated signaling decreased behavioral impulsivity and facilitated extinction learning (**Box 4**). The hypocretin/orexin system is a very promising target, but further work is required to fully exploit the orexin system for the treatment of psychostimulant-induced cognitive deficits.

### Brain-Derived Neurotrophic Factor (BDNF)

BDNF, a neurotrophin, is extensively distributed in the brain (259). BDNF plays a role in psychostimulant-induced behavioral effects. Inhibition of BDNF signaling and/or decreased expression of BDNF attenuated the rewarding effects of cocaine and cocaine-seeking behaviors (260–262). Methamphetamine withdrawal was associated with elevated BDNF levels in the dorsal striatum (263). In addition, genetically induced depletion of BDNF expression resulted in social cognitive deficits after chronic methamphetamine treatment compared with controls (264). Impaired BDNF signaling in the frontal and striatal regions during nicotine withdrawal was also associated with cognitive deficits (265). Together, the data suggest that decreased BDNF signaling possibly mediates psychostimulant-induced cognitive deficits.

Importantly, increase in BDNF signaling plays a role in consolidation of both recognition and spatial memory (266). Intracerebroventicular injection of antibodies to BDNF attenuated spatial learning in rats (267). Increase in BDNF signaling was also associated with extinction of fear-associated memories (268, 269). Interestingly, infusing BDNF into the ventral hippocampus increased the firing rate of neurons in the infralimbic PFC, which plays an important role in extinction learning (270). Importantly, increased BDNF signaling *via* stimulating tropomyosin-related kinase B (Trk B) receptors in the infralimbic PFC facilitated extinction of cocaine-induced CPP (112) (**Table 1**; **Figure 2**). Also, the study showed that systemic administration of Trk B receptor agonist facilitated extinction of cocaine-associated memories (**Table 3**). Overall, receptors mediating BDNF signaling are promising targets for facilitation of extinction of drug-associated memories and could be used for advancing treatment of psychostimulant addiction (**Box 4**). However, further work is required to understand BDNF signaling in specific circuits to maximally exploit its receptors as a therapeutic target.

### Corticotrophin Releasing Factor (CRF) Receptors

The role of CRF receptors in the behavioral and rewarding effects of psychostimulants has been previously reviewed (271–273). Blockade of CRF1 and CRF2 receptors in the VTA attenuated the reinforcing effects of cocaine (274). Interestingly, the rewarding effects of cocaine were enhanced in mice lacking CRF1 receptors compared with wild-type controls (275). Importantly, chronic cocaine administration induced memory deficits in wild-type mice but not in CRF1 deficient mice (276). In addition, cocaine withdrawal-induced memory deficits were observed in CRF2-deficient mice compared with wild-type controls (277). These data suggest that CRF receptors mediate cocaine withdrawal-induced impairment of memory and other cocaine-dependent effects.

CRF receptors are extensively distributed in brain regions that play a role in learning and memory (278). Blockade of CRF receptors, using CRF antagonist d-Phe-CRF, improved cognitive performance (279). Further, the study also showed that blockade of CRF1 receptors using CRF1 selective antagonist NBI 35965 improved memory in PFC-dependent tasks. Taken together, the data suggest that CRF receptors can be targeted to alleviate psychostimulant-induced memory deficits.

Importantly, CRF receptors in the VTA play a role in reinstatement of cocaine seeking (280). In addition, the same study showed that after cocaine self-administration and extinction training, stimulation of CRF2 receptors in brain slices resulted in increased glutamate release and decreased GABA release as compared in cocaine-naïve animals. These data suggest that extinction training and cocaine exposure altered CRF2-mediated transmission. Importantly, infusions of the CRF receptor antagonist α-helical CRF(9-41) into the basolateral amygdala enhanced extinction of fear-associated memories (281). However, further work needs to be carried out to determine if CRF receptors play a role in extinction of psychostimulant drug-associated memories. In summary, CRF receptors could serve as a potential target to alleviate psychostimulant-induced memory deficits and/or promote extinction of drug-associated memories.

## Non-Pharmacological Approaches for Treatment of Psychostimulant-Induced Cognitive Deficits

### Brain Stimulation

Brain stimulation can be achieved using a variety of different approaches such as transcranial magnetic stimulation, deep brain stimulation (DBS) using intracranial electrodes, transcranial direct current stimulation, and vagus nerve stimulation (282). Evidence from both human and animal studies supports use of brain stimulation to ameliorate cognitive deficits and improve learning and memory. For example, in humans, increase in verbal working memory accuracy was observed following transcranial magnetic stimulation (283). Similarly, DBS of the ventromedial PFC resulted in improvement in novel object recognition memory compared with that in controls in animals (284). Intracranial DBS also improved spatial memory in rats as assessed using the Morris water maze task (285). Also, DBS facilitated extinction of fear-associated memories (286, 287).

More importantly, DBS using intracranial electrodes attenuated reinstatement of cocaine seeking (288). Also, low-frequency DBS, but not high-frequency DBS, of the ventral striatum strengthened extinction of morphine-associated memories in rats (289). In addition, low-frequency stimulation of the ventral striatum was accompanied by an increase in immediate early gene c-fos synthesis in brain regions associated with extinction such as the infralimbic PFC and amygdala, suggesting increased activity of these regions. Importantly, vagal nerve stimulation during extinction training improved rates of extinction and reduced reinstatement of cocaine seeking in rats (151) (**Table 3**). Interestingly, DBS of subthalamic nucleus and vagal nerve stimulation also helped in decreasing decisional impulsivity in “risk preferring” rats compared with controls (290, 291).

Together, these data suggest that brain stimulation can help in both decreasing impulsivity and facilitating extinction of psychostimulant-associated memories. Thus, brain stimulation has the potential to alleviate multiple cognitive deficits. Future work must focus on identifying precise neural substrates and brain stimulation parameters to fully exploit the benefits of brain stimulation in psychostimulant addiction treatment. Furthermore, identification of pharmacological compounds that will help in improving efficacy of brain stimulation in addiction treatment will also be very useful.

### Exercise

Exercise in animals influences psychostimulant-dependent behavioral effects. For example, exercise attenuated reinstatement of cocaine seeking after a period of abstinence (292, 293). In addition, reinstatement of cocaine seeking in high impulsive rats was attenuated when animals were treated with a combination of atomoxetine and exercise during withdrawal from cocaine compared with either treatment alone (294). Importantly, post-extinction exercise training was more effective than extinction alone in attenuating reinstatement of cocaine seeking (295).

Exercise in the form of wheel running and swimming has been shown to improve learning and memory (296). Consistent with these findings, exercise using a treadmill attenuated morphine withdrawal-induced memory deficit in rats (297). Also, exercise facilitated extinction of fear-associated memories (298, 299). However, it is not known if exercise facilitates extinction of psychostimulant-associated drug memories. Further, effects of exercise on amelioration of psychostimulant withdrawal-associated memory deficits have not been explored. Several questions such as intensity and duration of exercise, neural changes as a consequence of exercise, and optimal combination of exercise with pharmacological medications need to be determined to use exercise most efficaciously as a tool for psychostimulant addiction treatment.

### Promoting Neurogenesis

Psychostimulant exposure impairs neurogenesis in the hippocampus in adult animals. For example, chronic exposure to nicotine, methamphetamine, and cocaine altered/blunted neurogenesis in the hippocampus (300–303). In addition, cocaine withdrawal-induced memory deficits were associated with blunted neurogenesis in the hippocampus (304). Hippocampal neurogenesis has been shown to play a role in consolidation of memory (305). Also, disruption of adult hippocampal neurogenesis impaired short- and long-term memory formation (306).

Relevant to this review, enhancing neurogenesis facilitated extinction of fear-associated memories (307, 308). Furthermore, pharmacological facilitation of neurogenesis facilitated extinction of morphine-associated memory (309). Importantly, increasing hippocampal neurogenesis in adult animals using chronic intracerebroventricular infusions of lysophosphatidic acid (LPA; an endogenous lysophospholipid with pro-neurogenesis effects) facilitated extinction of cocaine-associated memories (310). In contrast, suppression of neurogenesis using cranial irradiation resulted in resistance to extinction of cocaine seeking (311). Together, the above data suggest that pharmacological manipulation of adult hippocampal neurogenesis could facilitate extinction of drug-associated memories (**Box 4**). In summary, promoting neurogenesis can serve as an important strategy to treat psychostimulant addiction. However, future research must focus on understanding cellular mechanisms that underlie psychostimulant-induced impairment of hippocampal neurogenesis and identify pathways that can promote neurogenesis. Together, both of the above-described approaches will help to effectively treat psychostimulant-induced cognitive deficits.

## Future Directions

### Ghrelin

Ghrelin is an orexigenic peptide hormone acting on receptors in both the brain and periphery (312). Modulation of ghrelin altered effects of psychostimulants. For example, administration of ghrelin enhanced the rewarding effects of cocaine (313). Consistent with these findings, blockade of ghrelin-mediated transmission attenuated behavioral effects of cocaine, amphetamine, and nicotine (314, 315). In cocaine-experienced animals, during early withdrawal, ghrelin levels were elevated possibly in anticipation of cocaine (316). Similarly, in abstinent smokers, elevated ghrelin levels were associated with increased craving and relapse (317).

More importantly, ghrelin is neuroprotective, promotes hippocampal neurogenesis, and enhances learning and memory (318–320). Elevation of ghrelin levels as a consequence of food deprivation facilitated extinction of fear-associated memories, possibly by inhibition of long-term depression in the lateral amygdala (321). Consistent with these findings, a human clinical study reported facilitated extinction of fear-associated memories in subjects that had increased ghrelin levels as a result of overnight fasting (322). Based on these data, it is hypothesized here that increasing ghrelin-mediated signaling during extinction training may facilitate extinction of drug-associated memories. However, experimental data supporting this hypothesis are currently lacking. Besides, the precise mechanism of how ghrelin facilitates learning still needs to be explored. Nevertheless, there exists strong rationale for assessing the effects of ghrelin in extinction of drug-associated memories. Finally, based on the above data, it appears that elevated ghrelin levels are associated with craving in abstinent drug-dependent individuals, facilitation of extinction learning, and neuroprotection/neurogenesis. It is possible that ghrelin in different brain regions may have a differential role. Future work may need to understand the role of ghrelin in specific brain circuitries to fully exploit the therapeutic potential of ghrelin.

### Oxytocin

Oxytocin is synthesized by hypothalamic nuclei such as the supraoptic, parvocellular, and accessory nuclei. Oxytocin-containing neurons from these nuclei primarily project to posterior pituitary, but they also innervate brain regions mediating reward and emotion such as the PFC and amygdala (323). Systemic administration of oxytocin attenuated reinstatement of cocaine and methamphetamine seeking (324, 325). Consistent with this study, direct injection of oxytocin in the NAcc attenuated methamphetamine-induced CPP (326). Together, these data suggest that activation of oxytocin receptors attenuated drug-associated memories. Additionally, cocaine withdrawal was associated with increased oxytocin receptor binding in the piriform cortex, lateral septum, and amygdala (327).

Oxytocin receptors are extensively found in the PFC (328). Interestingly, activation of oxytocin receptors in the infralimbic PFC facilitated extinction of fear-associated memories (329, 330). Further social cues, such as presence of an animal, during extinction learning increased PFC oxytocin transmission (330). Overall, the data suggest that oxytocin receptor activation in the PFC facilitated extinction learning. However, the effects of increased oxytocin transmission on extinction of drug-associated memories have not been investigated. Together, these findings suggest that changes in oxytocin transmission may mediate some of the emotional and cognitive deficits associated with cocaine use. Based on the above-described findings, oxytocin receptors may serve as useful targets for the treatment of psychostimulant addiction, especially in promoting extinction of drug-associated memories (**Box 4**).

### Vasopressin

Vasopressin and its receptors play a role in psychostimulant-dependent behavioral effects. For example, elevated levels of vasopressin mRNA in the amygdala were observed in animals during withdrawal from cocaine (331). Additionally, blockade of vasopressin 1a receptors in the NAcc during conditioning attenuated expression of cocaine-induced CPP. Blockade of vasopressin 1b receptor also attenuated reinstatement of methamphetamine-induced CPP (332). Finally, blockade of vasopressin 1a receptors reversed oxytocin-induced attenuation of reinstatement of methamphetamine seeking (325). Together, the above evidence suggests a role for vasopressin in cocaine- and methamphetamine-dependent behavioral effects.

Vasopressin neurons and receptors are extensively found in brain regions involved in learning and memory such as the hippocampus, PFC, and amygdala (333–335). Knockout of vasopressin 1b receptor impaired hippocampal-dependent memory tasks (336). Vasopressin also plays an important role in social memory (337). Furthermore, blockade of vasopressin 1b receptor attenuated stress-induced impairment of memory (338). Elevated levels of vasopressin mRNA in the amygdala were also reported in animals showing high predisposition to stress-induced reinstatement of heroin seeking (339). A recent study has suggested that vasopressin may be involved in risky behaviors in humans, which suggest that it may have a role in impulsivity (340). In summary, the above data suggest that vasopressin-mediated neurotransmission is involved in memory and drug-dependent effects. Although still early, vasopressin receptors may serve as targets for treatment of psychostimulant-induced cognitive deficits.

### Protein Kinase Cε

PKCɛ is extensively found in the brain and is a downstream mediator of G-protein receptor signaling (341). Recent studies suggest that PKCɛ possibly mediates the reinforcing effects of psychostimulants like nicotine and cocaine. For example, mice lacking PKCɛ showed reduced mRNA levels of α6 and β3 nAChR subunits in brain regions associated with drug reward such as the VTA and striatum (342). Consistent with these findings, knockout of PKCɛ reduced nicotine-induced CPP and attenuated nicotine self-administration compared with wild-type controls. Relevant to this review, the infralimbic PFC showed elevated levels of PKCɛ after with withdrawal from extended cocaine self-administration experience (343). More importantly, inhibition of PKCɛ in the infralimbic PFC attenuated reinstatement of cocaine seeking. However, the effects of PKCɛ expression in the infralimbic PFC on extinction learning have not been assessed.

Activation of PKCɛ facilitates learning and memory (344, 345). In fact, inhibition of PKCɛ using peptides that directly bind to PKCɛ attenuated recognition memory as assessed using novel object recognition task (345). It is postulated that the memory-enhancing effects of PKCɛ activation are mediated *via* increased activity of ERK1/2 in the hippocampus. Together, the above data suggest that activation of PKCɛ could be useful in facilitating extinction of drug-associated memories. Based on the role of PKCɛ in memory and cocaine-dependent behaviors, it is hypothesized that PKCɛ may be an attractive target for treating psychostimulant addiction by promoting extinction learning.

### Peroxisome Proliferator-Activated Receptor γ (PPARγ) Receptors and Insulin

Insulin and PPARγ agonists influence the behavioral and psychological effects of drugs of abuse. For example, a recent double-blind randomized study reported that patients receiving PPARγ agonist pioglitazone compared with placebo reduced cocaine craving and improved brain white matter integrity in cocaine-dependent patients (346). In animals with cocaine self-administration experience, insulin levels were reduced by approximately 40–70% during cocaine self-administration (316). In addition, intra-VTA injections of insulin attenuated cocaine-induced increase in NAcc dopamine and decreased cocaine-induced increase in locomotor activity (347).

Additionally, insulin and PPARγ agonists play a role in alleviating memory deficits. For example, systemic administration of insulin and insulin-growth factor 2 (IGF-2) facilitated learning and memory (348, 349). Additionally, intranasal insulin administration improved memory in patients with either mild cognitive impairment or early Alzheimer’s disease (350). Further, PPARγ agonists improved memory in some humans with early Alzheimer’s disease (351). These memory-enhancing effects of PPARγ agonists are possibly mediated by actions of PPARγ agonists on hippocampal dentate neurons (352, 353). In summary, both insulin and PPARγ play a role in cognition and memory and could influence the behavioral effects of psychostimulants.

Importantly, PPARγ agonist pioglitazone attenuated alcohol-induced spatial memory deficit as assessed using the Morris water maze (354). Additionally, pioglitazone attenuated drug-induced heroin seeking (355). Finally, increased IGF-2-mediated transmission in the hippocampus facilitated extinction of fear-associated memories (356). It is hypothesized that this IGF-2-mediated facilitation of extinction occurs *via* stimulation of neurogenesis (357). However, it is not known if insulin and PPARγ agonists could facilitate extinction of drug-associated memories? Could insulin and PPARγ agonists be used to ameliorate psychostimulant withdrawal-induced memory impairment? Future work will need to address these and other questions.

### Enzymes Involved in Epigenetic Changes

Epigenetic changes occur as a consequence of behavioral activity, learning, and/or drug exposure (358). In fact, enzymes involved in epigenetic DNA changes are involved in psychostimulant and non-psychostimulant drug-associated memories. For example, DNA methylation *via* chronic l-methionine (MET) attenuated reinstatement of cocaine seeking (359). Also, knockdown of histone methyltransferase PR containing domain 2 (PRDM2) in the dorsomedial PFC using viral vectors enhanced stress-induced reinstatement of alcohol seeking (360). Genetically induced loss of histone acetyltransferase CREB-binding protein (CBP) in the NAcc attenuated cocaine-induced CPP.

Activity-dependent epigenetic changes play an important role in learning and memory consolidation (361). Importantly, the enzymes that mediate these epigenetic changes could be targeted to facilitate learning and memory. For example, blocking of histone deacetylase (HDAC3) enzyme activity in the dorsal hippocampus enhanced long-term memory for object location (114). Additionally, manipulation of enzymes involved in epigenetic changes facilitated extinction learning. For example, inhibition of histone acetyltransferase (HAT) p300 enzyme, which is highly expressed in pyramidal neurons of the infralimbic PFC, facilitated extinction of fear-associated memories (362). Importantly, blocking of HDAC3 deacetylase activity in the dorsal hippocampus, but not the infralimbic PFC, facilitated extinction of cocaine-associated memories (114) (**Table 1**; **Figure 2**). Overall, these data suggest that enzymes involved in epigenetic changes could play a role in facilitation of extinction of psychostimulant-associated memories. More generally, they could also play a role in the treatment of cognitive deficits associated with psychostimulants such as impulsivity and memory impairments. However, much work remains to not only identify specific enzymes but also to identify specific brain regions where these enzymes are actively involved in psychostimulant-induced cognitive deficits.

### MicroRNAs (miRs)

The role of non-coding microRNAs (miRs) has been implicated in psychostimulant-dependent behaviors. For example, methyl CpG binding protein 2 (MeCP2) and miR-212 in the dorsal striatum play a role in regulating escalation of cocaine intake in rats with extended access to cocaine (363). Further, upregulation of miR-212 and miR-132 in the dorsal striatum persisted for approximately 10 days after withdrawal of cocaine (364). Similarly, miR-496-3p, miR-194-5p, miR-200b-3p, and miR-181a-5p were upregulated significantly following methamphetamine exposure (365). Together, the data suggest that exposure to psychostimulants alters expression of microRNAs.

The role of non-coding miRs has been implicated in cognitive processes such as impulsivity, learning, and memory. For example, several miRs in the amygdala such as miR-190b, miR-28a, miR-340, miR-219a, and miR-491 have been reported to correlate with inhibitory control (366). Thus, theoretically decreased expression of these miRs could result in impulsive behaviors, although direct experimental evidence for this hypothesis is currently lacking. Similarly, miR-641, which binds to SNAP-25 gene, has been implicated in impulsive behaviors (367). In addition, miR-183-96-182 has been associated with comorbid ADHD and drug addiction (368). Together, these data suggest that miRs play a role in regulating impulsive behavioral traits.

miRs also play a role in memory (369). For example, inhibition of miR-9-3p resulted in deficits in hippocampal-dependent tasks (370). Overexpression of miR-144-3p in the basolateral amygdala facilitated extinction of fear-associated memories in C57BL/6 mice (371). In addition, the same study showed that overexpression of miR-144-3p in the basolateral amygdala rescued extinction of fear memories in S1 mice, which show resistance to extinction of fear memories. Similarly, extinction training after fear conditioning trials resulted in increase in expression of miR-128b in the infralimbic PFC, and overexpression of miR-128b in the infralimbic PFC facilitated extinction of fear-associated memories (372). Importantly, significant increases in the expression of miR-101b, miR-137, miR-212, and miR-132 in NAcc shell and miR-137 in the dorsal striatum were observed after extinction training and reinstatement of cocaine seeking in rats (373). Future studies must focus on brain regions associated with extinction learning such as the basolateral amygdala and infralimbic PFC to identify miRs that are involved in extinction of drug-associated memories. Although currently data are lacking, based on the above data, non-coding miRs could be targeted to facilitate extinction of drug-associated memories and to reduce psychostimulant-associated impulsivity.

### Gender and Sex Gonadal Hormones

Both gender and sex gonadal hormones influence cognition. For example, behavioral impulsivity was greater in males compared with females (374). In contrast, females compared with males showed more decisional impulsivity, preferring small immediate rewards compared with larger delayed rewards. Treatment with progesterone attenuated decisional impulsivity for food reward in both males and females (375). Interestingly, progesterone alone attenuated both behavioral and decisional impulsivity for cocaine rewards in female but not male rats (141, 142) (**Table 2**). These data suggest that sex gonadal hormones influence impulsive behaviors. Also, amphetamine worsened impulsive behavior in females compared with males (376). Together, the above data suggest that gender and sex gonadal hormones influence psychostimulant-induced impulsive behaviors.

With respect to extinction of fear-associated memories, differential electrophysiological responses in the infralimbic and prelimbic PFC have been reported between males and females. For example, female rats compared with male rats showed persistent activity in the prelimbic PFC during extinction training, and there was lack of activity in the infralimbic PFC during extinction recall (377). Additionally, the role of estrogen and progesterone in extinction of fear-associated memories has been evaluated. In ovariectomized female rats, estrogen alone or in combination with progesterone facilitated extinction of fear-associated memories (378). Several other studies support the role of estrogen in extinction of fear-associated memories (379, 380). Together, the data suggest that gender and sex gonadal hormones may influence extinction learning.

Gender and sex gonadal hormones also influence psychostimulant drug-associated memories. Extinction of cocaine-induced CPP took longer in male compared with female adolescent rats (110). More recent work has shown that after similar extinction training, context-induced reinstatement of methamphetamine seeking was more pronounced in male compared with female rats (381). Further, the study showed that this difference in methamphetamine seeking between male and female rats was possibly mediated by differential plasticity in the dentate gyrus in the hippocampus. Together, the data suggest differential gender-dependent responses to extinction of psychostimulant drug-associated memories. Treatment with 17β estradiol compared with controls facilitated extinction of cocaine-induced CPP in female rats (150) (**Table 3**). Allopregnanolone, a steroid synthesized from progesterone, attenuated reinstatement of drug-induced cocaine seeking in female but not male rats (382). Allopregnanolone also attenuated reinstatement of cocaine seeking in low impulsive female rats but not in high impulsive female rats, classified as such on baseline performance prior to cocaine exposure (383). However, further studies are required to fully exploit the role of estrogen and progesterone in facilitation of extinction of psychostimulant drug-associated memories. In summary, the above data suggest that gender and sex-gonadal hormones could play an important role in cognitive deficits associated with psychostimulant drugs. However, further work is required to develop more efficacious gender-based treatments for cognitive deficits in human drug-dependent subjects.

## Conclusion

Addiction to psychostimulant drugs continues to be a challenge, and current treatment options available for psychostimulant addiction are not adequate. Targeting cognitive deficits in patients dependent on psychostimulants provides an excellent opportunity to improve retention and clinical outcomes of addiction treatment programs. Cognitive deficits should especially be targeted in psychostimulant-dependent patients with a history of prenatal drug exposure and patients with comorbid psychiatric disorders known to be associated with cognitive deficits. In this review, several neural substrates mediating psychostimulant-induced cognitive deficits and identified using preclinical animal models have been discussed. It remains to be seen if these could be translated into viable pharmacological targets for medications to be used in humans to improve clinical outcomes of patients dependent on drugs of abuse. However, the main question is which of the described targets would be most ideal to carry forward into the clinic. Among the various targets described, it will be important to focus on targets that could help alleviate multiple psychostimulant-induced cognitive deficits such as impulsivity and memory impairment (e.g., orexin and cannabinoid receptors). Further, drugs that facilitate/strengthen extinction of drug-associated memories should be an essential strategy of addiction treatment programs.

Future studies must focus on identifying specific circuits mediating psychostimulant-induced cognitive deficits. Better understanding of the role of non-coding miRs, neurogenesis, and enzymes involved in epigenetic changes will greatly help in developing highly selective treatments. Finally, combining non-pharmacological strategies such as brain stimulation and exercise with pharmacological compounds will enhance alleviation of psychostimulant-induced cognitive deficits. In this review, the focus has been on targeting specific psychostimulant-induced cognitive deficits such as impulsivity and impairment of learning/memory. However, psychostimulant-induced cognitive deficits include other deficits such as impairment in attention, lack of cognitive flexibility, and impaired decision making, which have not been discussed in this review but need to be therapeutically addressed. In conclusion, a multipronged strategy targeting behavioral, emotional, and cognitive deficits in recovering abstinent addicts will greatly improve outcomes of psychostimulant addiction treatment.

## Author Contributions

This paper was conceived, researched, and written by MD’S.

## Funding

This work was supported by Bower, Bennet, and Bennet Endowed Chair Research Award awarded to MD’S by The Raabe College of Pharmacy, Ohio Northern University (ONU), Ada, Ohio.

## Conflict of Interest Statement

The author declares that the research was conducted in the absence of any commercial or financial relationships that could be construed as a potential conflict of interest.

## Abbreviations

ACPC, 1-aminocyclopropanecarboxylic acid; AMPA, amino-3-hydroxy-5-methyl-4-isoxazolepropionate/kainate; AP-5, (2*R*)-amino-5-phosphonovaleric acid; AQP-4, aquaporin-4; CDPPB, 3-cyano-*N*-(1,3-diphenyl-1*H*-pyrazol-5-yl)benzamide; CPP, conditioned place preference; 5-CSRTT, 5-choice serial reaction time task; DBS, deep brain stimulation; DDT, delay discounting task; 5HT, serotonin; GABA, γ-aminobutyric acid; GLT, glutamate transporter; MDMA, 3,4-methylenedioxymethamphetamine; MK-801, (5*R*,10*S*)-(−)-5-methyl-10,11-dihydro-5*H*-dibenzo[*a*,*d*]cylcohepten-5,10-imine; mPFC, medial prefrontal cortex; mGlu, metabotropic glutamate; MPEP, 2-methyl-6-(phenylethynyl)pyridine; MTEP, 3-(2-methyl-1,3-thiazol-4-yl)ethynyl)pyridine; mRNA, microRNA; MOR, mu opioid receptor; NAcc, nucleus accumbens; NMDA, *N*-methyl-d-aspartate; OFC, orbitofrontal cortex; PAMs, positive allosteric modulators; Trk B, tropomyosin-related kinase B; VTA, ventral tegmental area; xCT, cystine–glutamate exchanger.
